# Dynamic infrared thermography in free flap surgery: A systematic review

**DOI:** 10.1016/j.jpra.2025.11.027

**Published:** 2025-11-27

**Authors:** Warre Clarys, Valentine Hotome, Stefan Hummelink, Simon Verspeek, Veronique Verhoeven, Wiebren A.A. Tjalma, Gunther Steenackers, Filip Thiessen

**Affiliations:** a University of Antwerp, InViLab Research Group, Groenenborgerlaan 171, Antwerp, B-2020, Belgium; b Faculty of Medicine and Health Sciences, University of Antwerp, Universiteitsplein 1, Antwerp, B-2610, Belgium; c Radboud University Medical Center, Department of Plastic Surgery, Geert Grooteplein Zuid 10, Nijmegen, 6525 GA, The Netherlands; d Department of Plastic, Reconstructive and Aesthetic Surgery, Multidisciplinary Breast Clinic, Antwerp University Hospital, University of Antwerp, Wilrijkstraat 10, Antwerp, B-2650, Belgium; e Department of Family Medicine and Population Health, Faculty of Medicine and Health Sciences, University of Antwerp, Doornstraat 331, Wilrijk, B-2610, Belgium; f Department of Obstetrics and Gynaecology, Multidisciplinary Breast Clinic, Gynaecological Oncology Unit, Antwerp University Hospital, University of Antwerp, Drie Eikenstraat 655, Edegem, B-2650, Belgium

**Keywords:** Dynamic infrared thermography, Free flap surgery, Perforator mapping, DIEP flap, Augmented reality, Flap monitoring

## Abstract

**Background:**

Dynamic infrared thermography (DIRT) is increasingly being utilized for perforator selection and perfusion assessment in reconstructive microsurgery, particularly in procedures such as deep inferior epigastric perforator (DIEP) flap reconstructions. This systematic review evaluates the current evidence regarding the diagnostic performance, clinical applicability, and recent advancements of DIRT in free flap surgery.

**Methods:**

A systematic search was conducted in accordance with PRISMA guidelines across the PubMed, Scopus, and Web of Science databases, including studies published up to December 2024. Inclusion criteria comprised primary studies employing DIRT in free flap surgery using a dynamic cold challenge, while static thermography experiments were excluded. Data on study characteristics, diagnostic accuracy, and recent technological developments were extracted and assessed using the QUADAS-2 tool.

**Results:**

A total of 31 studies were included, ranging from case reports to prospective cohort studies. DIRT demonstrated sensitivities between 84 % and 95 % for perforator detection, comparable to computed tomography angiography (CTA) and superior to hand-held Doppler (HHD). Despite limitations such as lower resolution and shallow detection depth, recent innovations, including augmented reality (AR), smartphone integration, and artificial intelligence (AI), offer promising solutions for enhanced reliability and user-friendliness.

**Conclusion:**

DIRT is an effective, non-invasive technique with diagnostic accuracy comparable to CTA for preoperative and intraoperative perforator selection in free flap surgery. Further standardization and large-scale clinical studies are warranted to establish DIRT as a standard diagnostic tool in reconstructive microsurgery.

## Introduction

The success of free flap surgery depends on selecting an optimal perforator with sufficient caliber and perfusion, as it supplies the entire flap after microsurgical anastomosis. Accurate perforator mapping enhances dissection precision and efficiency, potentially reducing operative time by up to 1 h.^1^, ^2^, ^3^, ^4^

Current imaging modalities for perforator mapping include computed tomography angiography (CTA), handheld Doppler (HHD), magnetic resonance angiography (MRA), and infrared thermography (IRT). CTA remains the gold standard but requires contrast and exposes patients to radiation.^5^ Alternatives such as color Doppler ultrasound (CDU) and MRA are limited by operator dependence and contrast use.

Dynamic infrared thermography (DIRT) has emerged as a noninvasive technique providing real-time functional imaging during skin rewarming after a cold challenge. Infrared cameras capture temperature distribution, with well-perfused perforators appearing as hotspots.^6^^,^^7^

Despite its advantages, DIRT has not replaced CTA because of limited depth, spatial resolution, and subjective interpretation. Earlier studies also lacked standardized cooling and analysis protocols, contributing to heterogeneity. Recent technological advances are improving both accuracy and usability.

Previous reviews discussed IRT broadly in plastic surgery,^8^ focused on abdominal breast reconstruction with mixed static and dynamic methods,^9^ or examined only smartphone-based IRT.^10^ This review specifically addresses dynamic IRT with a cold challenge across free-flap types, integrating recent advances in AR, AI, and simulation, and updating comparative accuracy data versus CTA, HHD, and ICG-FA.

## Methodology

This systematic review followed the Preferred Reporting Items for Systematic Reviews and Meta-Analyses (PRISMA) guidelines, including the Diagnostic Test Accuracy (PRISMA-DTA) extension,^11^ with a predefined protocol specifying objectives, inclusion criteria, and analytical methods. The study was registered in the PROSPERO database (CRD42024586929).

### Search strategy

A systematic search was conducted in PubMed, Scopus, and Web of Science for studies published up to December 5, 2024, using the search string: (DIEP OR free flap OR perforator OR breast reconstruction OR mammoplasty) AND (thermography OR infrared OR DIRT) NOT (near infrared OR spectroscopy), limited to Title/Abstract fields. Reference lists of key studies and reviews were screened manually. No language limits were applied, but an English abstract was required.

### Eligibility criteria

#### Study designs

Eligible studies included all primary research on DIRT in free or perforator flap surgery, prospective or retrospective studies, case series/reports, commentaries, technical notes, and relevant animal work. Reviews and meta-analyses were excluded to focus on primary data. Studies using only static IRT without dynamic cooling or lacking full-text access were excluded. Randomized trials were eligible, though none were identified.

#### Population

Included studies involved patients, animals, or volunteers undergoing perforator mapping with DIRT. While the main focus was breast reconstruction (e.g., DIEP flaps), other donor sites (e.g., anterolateral thigh (ALT), muscle flaps) were considered. Studies limited to pedicled flaps were excluded unless free-flap data were separately extractable.

#### Intervention

The intervention of interest was DIRT applied during any perioperative phase, preoperative planning, intraoperative assessment, or postoperative monitoring. Studies using adjunctive modalities such as fluorescent angiography or HHD in conjunction with DIRT were included to compare diagnostic performance.

### Reference standard

Reference standards comprised CTA preoperatively, HHD and surgical inspection intraoperatively, and clinical monitoring postoperatively.^12^^,^^13^ DIRT was evaluated relative to these established modalities.

### Study selection and data extraction

Two reviewers independently screened titles/abstracts and full texts, resolving disagreements by discussion. Extracted data included author, year, design, sample size (subjects and/or flaps), flap type, DIRT protocol (camera, cooling, image analysis), comparator modalities (CTA, HHD, ICG-FA), key findings on perforator detection, and reported accuracy measures. Information on innovative techniques (AR integration, machine-learning algorithms, or novel imaging devices) was also extracted when applicable. Where relevant, contextual comparisons were drawn from excluded reviews offering additional insight.

### Quality and bias assessment

Two reviewers independently assessed risk of bias and applicability using the QUADAS-2 tool,^14^ evaluating four domains: (1) Patient Selection, (2) Index Test, (3) Reference Standard, and (4) Flow/Timing. Each domain was rated as low, high, or unclear risk. Applicability concerns were assessed similarly for the first three domains. Discrepancies were resolved by discussion. Results are summarized in Table 2 and Figure 4. No studies were excluded based on quality alone.

## Results

### Study selection

**Figure 1:**
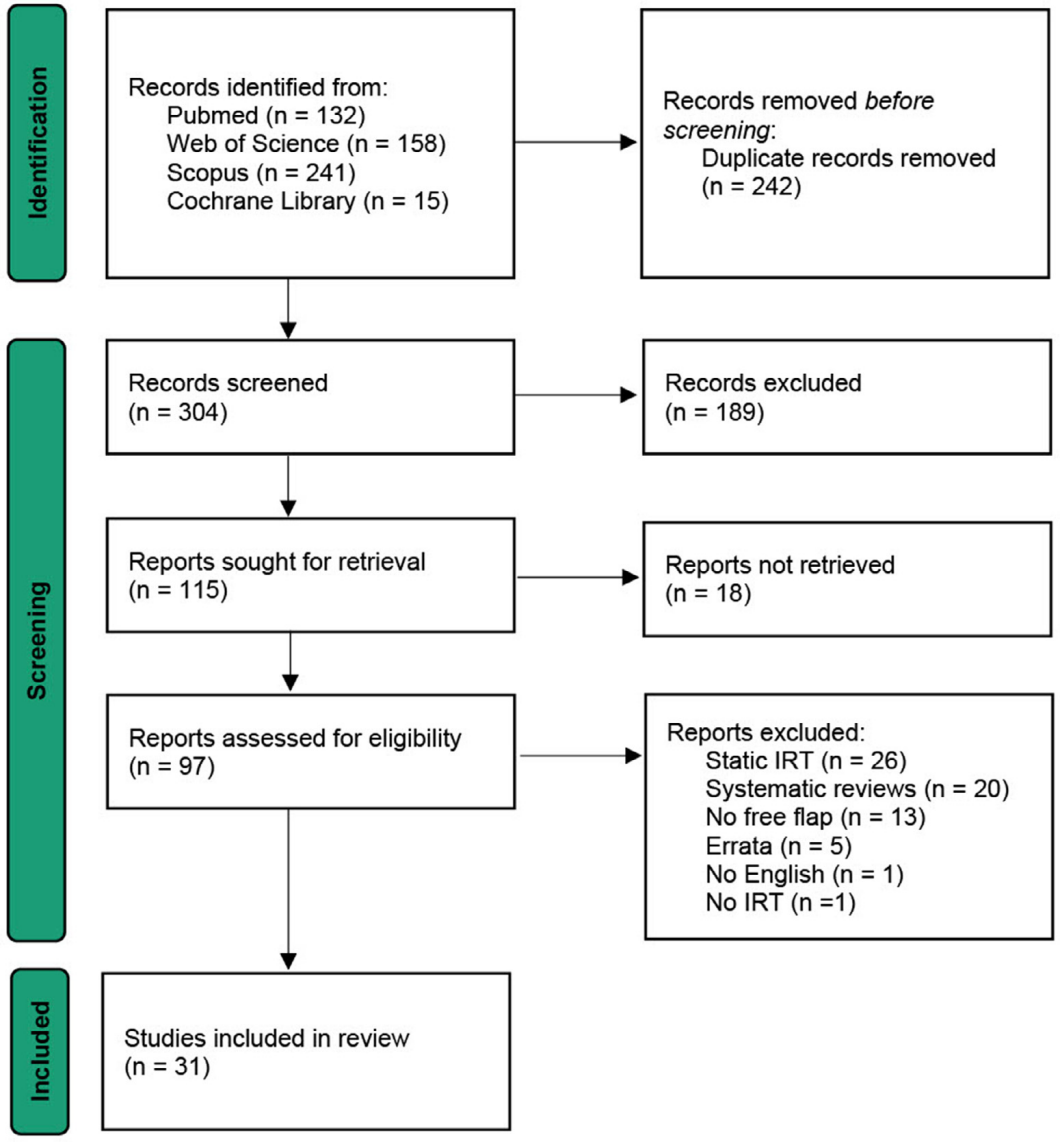
PRISMA flow diagram of the screening and selection process.

The initial literature search identified 304 records after duplicate removal. Title and abstract screening excluded 189 records due to being unrelated to DIRT or perforator flap imaging. Of 115 full-text articles sought, 18 were unavailable due to lack of full-text access or a non-English full-text version. About 97 articles were assessed for eligibility; Of these, 67 were excluded for the following reasons: static IRT only (n=26), secondary sources such as systematic reviews without original data (n=20), absence of free flap procedures (n=13), errata or corrections (n=5), lack of English abstract (n=1), and no use of IRT (n=1). 31 studies met all inclusion criteria and were included. The PRISMA flow diagram is shown in Figure 1.

### Study characteristics

Table 1 summarizes key characteristics of the 31 included studies, including authorship, journal, study population (human or animal), and main findings on DIRT in free flap surgery. Study designs ranged from patient series and feasibility studies to animal experiments and comparative evaluations. Sample sizes ranged from small early case series to larger recent cohorts such as Ismail et al.^15^

**Table 1:** Overview of the key characteristics of the included studies.

Study	Title	Study Type	Journal	Subjects	Key Findings
Chaudhry et al.^16^	In vivo perforasome perfusion in hemi-DIEP flaps for breast reconstruction: a validation study of dynamic infrared thermography	Clinical study	Plastic and Reconstructive Surgery - Global Open	15 patients	IRT enables non-invasive visualization of perforasome perfusion, optimizing perforator selection and flap design. It can complement or replace conventional mapping methods.
Chubb et al.^17^	Images in plastic surgery: digital thermography—a simple, reproducible means of preoperative planning and postoperative monitoring in perforator flaps	Technical note	Annals of Plastic Surgery	Not applicable	Thermal imaging is a simple, non-invasive, and cost-effective tool for identifying dominant perforators preoperatively and assessing flap perfusion postoperatively.
Chubb et al.^18^	True and 'choke' anastomoses between perforator angiosomes: part II. Dynamic thermographic identification	Clinical study	Plastic and Reconstructive Surgery	Nine patients	DIRT differentiates true and choke interperforator anastomoses and quantifies rewarming patterns following a logarithmic curve.
Dagdelen et al.^19^	A practical tip for intraoperative perforator vessel selection: portable thermal imaging	Case report, technical note	Turkish Journal of Plastic Surgery	One patient	Portable thermal imaging is a low-cost, accessible intraoperative aid for selecting dominant perforators in freestyle flaps.
de la Hoz et al.^20^	Automated thermographic detection of blood vessels for DIEP flap reconstructive surgery	Clinical study	International Journal of Computer Assisted Radiology and Surgery	27 time-lapse thermograms	Automated IRT accurately identifies perforators in real time with dice scores up to 0.9, reducing subjectivity in perforator selection.
de Weerd et al.^21^	Intraoperative dynamic infrared thermography and free-flap surgery	Prospective, clinical study	Annals of Plastic Surgery	10 patients	Intraoperative DIRT reliably detects arterial inflow and occlusion, often before clinical signs appear.
de Weerd et al.^22^	Perfusion dynamics of free DIEP and SIEA flaps during the first postoperative week monitored with dynamic infrared thermography	Clinical study	Annals of Plastic Surgery	20 patients	Perfusion progresses faster in the subdermal plexus than in subcutaneous tissue. Midline choke vessels form higher resistance zones.
de Weerd et al.^23^	The perfusion code of DIEP and MS-TRAM flaps is a hard nut to crack	Letter to other article	Journal of Plastic, Reconstructive and Aesthetic Surgery	Not applicable	Reducing flap size intraoperatively may discard tissue that later becomes adequately perfused; flap volume remains crucial for symmetry.
Hallock et al.^24^	Dynamic infrared thermography and smartphone thermal imaging as an adjunct for pre-operative, intraoperative, and postoperative perforator free flap monitoring	Technical note	Plastic and Aesthetic Research	Not applicable	Smartphone IRT has a short learning curve and supports safe flap design, intraoperative assessment, and postoperative monitoring.
Ismail_et al.^15^	Smartphone thermal imaging for preoperative perforator mapping in perforator based flaps	Clinical study	Cureus Journal of Medical Science	143 patients	IRT detected 68.5 % of perforators versus 75.5 % visually, with 83.2 % accuracy, showing good sensitivity and predictive value.
Itohet al.^25^	Use of recovery-enhanced thermography to localize cutaneous perforators	Clinical study	Annals of Plastic Surgery	12 patients	Recovery-enhanced IRT effectively visualizes perforators, outperforming Doppler in mapping multiple vessels, though equipment is bulky and costly.
Kimet al.^26^	An experimental and clinical study of flap monitoring with an analysis of the clinical course of the flap using an infrared thermal camera	Animal study	Bioengineering,	28 rats, 22 patients (eight free flaps)	No significant temperature differences were observed between pedicled and free flaps across all time points.
Ko et al.^27^	Detection of perforators using smartphone thermal imaging	Letter to article (Hardwicke et al.^28^)	Plastic and Reconstructive Surgery	Eight patients	FLIR ONE detects hotspots inconsistently but identifies more perforators than HHD, with fewer false positives from deeper vessels.
Liu et al.^29^	Reconstruction of thermographic signals to map perforator vessels in humans	Clinical study	Quantitative Infrared Thermography Journal	Five patients	Themographic signal reconstruction enhances vessel contrast and enables accurate perforator mapping after short occlusion periods.
Meier et al.^6^	Projected augmented reality in DIEP flap breast reconstruction: projecting perforators on the skin using dynamic infrared thermography	Clinical study	Journal of Plastic, Reconstructive and Aesthetic Surgery	71 patients	DIRT hotspots correlated with HHD in 97.3 % of cases and with CTA in 74.5 %, confirming DIRT's reliability for intraoperative projection.
Moderhak et al.^30^	Comparison of the exponential thermal transient parameterization methods with the simplified magnitude-temporal parametrization (SMTP) method in the unipedicled DIEP flap computer modelling and simulation	Simulation study	Quantitative Infrared Thermography Journal	One patient	SMTP modeling accurately detects perforators with high spatial contrast and low computational complexity, improving perfusion simulations.
Moderhak et al.^31^	Active dynamic thermography method for TRAM flap blood perfusion mapping in breast reconstruction	Clinical study	Quantitative Infrared Thermography Journal	38 patients	DIRT provides real-time, non-invasive TRAM flap perfusion mapping with high sensitivity and robustness to non-uniform thermal excitation.
Muntean et al.^32^	Using dynamic infrared thermography to optimize color Doppler ultrasound (CDU) mapping of cutaneous perforators	Animal study	Medical Ultrasonography	10 pigs	Combined DIRT+CDU shortened examination time and improved dominant perforator identification compared with CDU alone.
Muntean et al.^1^	Dynamic infrared mapping of cutaneous perforators	Technical note	Journal of Xiangya Medicine	Not applicable	DIRT serves as an accurate, reproducible mapping tool comparable to CTA and HHD, without their limitations.
Nergård et al.^33^	Internal mammary vessels' impact on abdominal skin perfusion in free abdominal flap breast reconstruction	Clinical study	Plastic and Reconstructive Surgery - Global Open	17 patients	Internal mammary vessel use alters abdominal perfusion patterns and may contribute to wound healing complications.
Niepel et al.^34^	Decision between contralateral and ipsilateral DIEP flap harvesting for unilateral breast reconstruction	Clinical study	European Journal of Plastic Surgery	17 patients	Faster rewarming occurred on the side with larger DIEA diameter, allowing targeted perforator dissection and shorter operative times.
Nischwitz et al.^35^	Thermal, hyperspectral and laser doppler. imaging: non-invasive tools for detection of the deep inferior epigastric artery perforators: a prospective comparison study	Clinical study	Journal of Personalized Medicine	18 patients	DIRI showed 94 % sensitivity and no false positives, outperforming hyperspectral imaging for perforator detection.
Pereira et al.^36^	TARDUS: a combined method for microsurgical easy planning with thermography, augmented reality and Doppler ultrasound	Clinical study	Journal of Plastic, Reconstructive and Aesthetic Surgery	15 patients	Combined thermography, AR, and Doppler (TARDUS) reduced perforator search time and improved intraoperative efficiency.
Sonda et al.^37^	Deep inferior epigastric perforator flap preoperative planning: a comparative analysis between dynamic infrared thermography, computerized tomography angiography, and hand-held Doppler	Clinical study	Microsurgery	12 patients	Sensitivity was 93.9 % for CTA, 69.0 % for HHD, and 92.1 % for DIRT; BMI showed no effect on perforator detection.
Thiessen et al.^38^	Dynamic infrared thermography (DIRT) in deep inferior epigastric perforator (DIEP) flap breast reconstruction: standardization of the measurement set-up	Technical note	Gland Surgery	Not applicable	Describes standardized DIRT setup using 3–5 °C. cooling for 3 minutes and serial imaging during pre-, intra-, and postoperative phases,
Thiessen et al.^39^	Dynamic infrared thermography (DIRT) in DIEP flap breast reconstruction: a clinical study with a standardized measurement setup	Clinical study	European Journal of Obstetrics & Gynecology and Reproductive Biology	33 DIEP flaps in 21 patients	Standardized DIRT confirmed CTA findings, detected intraoperative issues, and monitored perfusion postoperatively.
Verstockt et al.^40^	DIEP flap breast reconstructions: Thermographic assistance as a possibility for perforator mapping and improvement of DIEP flap quality	Pilot study	Applied Optics	Not specified	DIRT provides real-time mapping of dominant perforators and vascular territories, improving flap design and reducing necrosis risk.
Weum et al.^41^	Evaluation of dynamic infrared thermography as an alternative to CT angiography for perforator mapping in breast reconstruction: a clinical study	Clinical study	BMC Medical Imaging	25 patients	DIRT hotspots matched arterial HHD sounds and CTA perforators; HHD yielded occasional false positives not confirmed intraoperatively.
Yassin et al.^42^	Uses of smartphone thermal imaging in perforator flaps as a versatile intraoperative tool: the Microsurgeon’s Third Eye	Clinical study	JPRAS Open	20 patients (17 free flap patients)	Intraoperative hotspots corresponded to dominant perforators; lack of rewarming indicated pedicle kinking or venous issues.
Zetterman et al.^7^	Effect of cooling and warming on thermo-graphic imaging of the perforating vessels of the abdomen	Clinical study	European Journal of Plastic Surgery	16 volunteers	Longer cooling (300 s) improved hotspot contrast 3.4-fold and prolonged visibility compared to shorter cooling.
Zhu et al.^43^	Value of the combination of a smartphone-compatible infrared camera and a handheld Doppler ultrasound in preoperative localization of perforators in flaps	Clinical study	Heliyon	22 patients	Combining FLIR ONE PRO with HHD increased PPV and sensitivity for perforator localization compared to FLIR alone.

**Figure 2:**
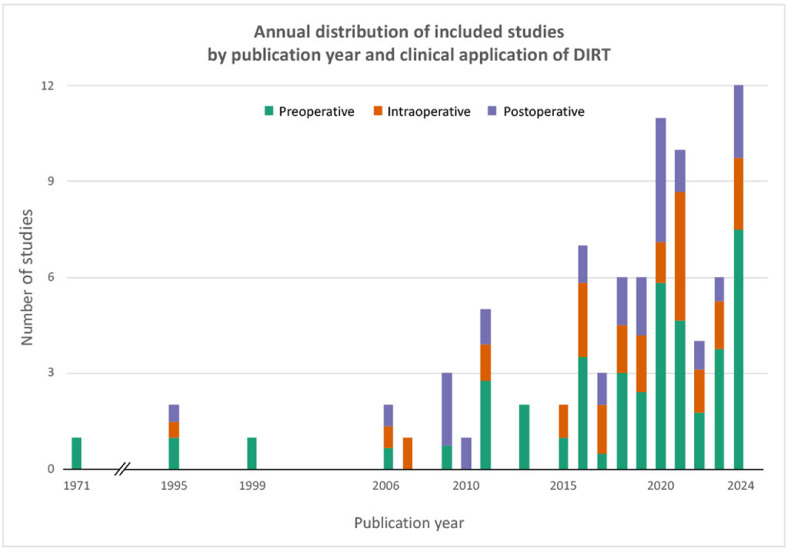
The figure illustrates a growing interest in DIRT over time, with a marked increase in preoperative applications in the last 5 years. Colors within each bar indicate the proportion of studies focused on preoperative mapping, intraoperative perfusion assessment, or postoperative monitoring. Note: This figure includes all studies identified during the literature search that involved IRT in the context of free flap surgery, including those excluded from the final review due to use of static IRT (n=26) or review format (n=20). As such, the figure reflects the broader research activity in the field rather than the final set of included studies alone.

Figure 2 shows the annual distribution of IRT-related publications, encompassing dynamic and static techniques and reviews, and demonstrates a clear upward trend. Preoperative DIRT studies now form over half of yearly reports, while intra- and postoperative applications such as perfusion assessment and flap monitoring have also increased, though less markedly.

**Figure 3:**
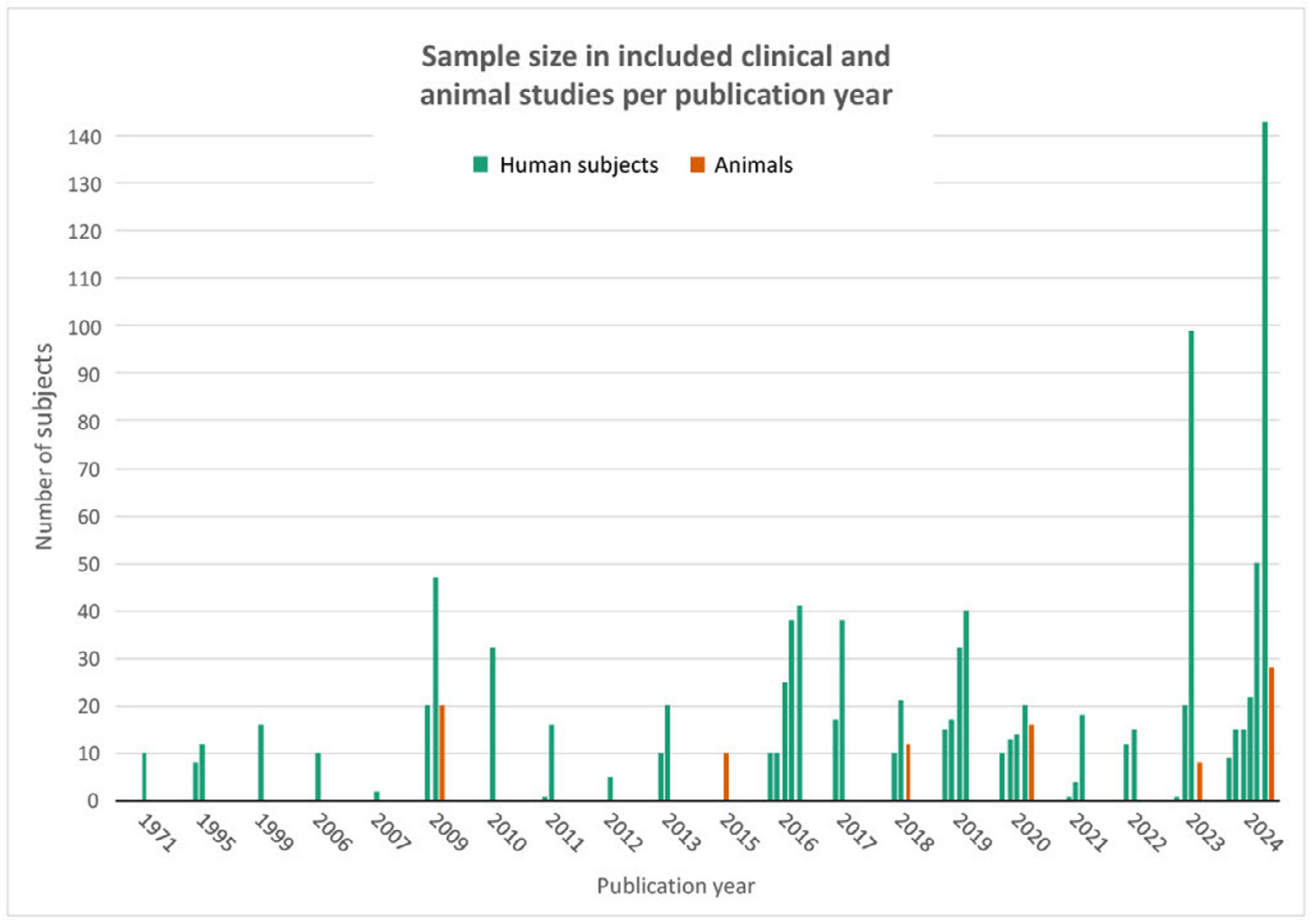
Sample sizes of clinical studies and animal studies per publication year. A notable increase in sample size can be seen in the last 5 years, with a set-back during the COVID-19 pandemic. Animal models remain to take up a small but consistent role in the provided literature across the study period. Note: This figure includes all studies identified during the literature search that involved IRT in the context of free flap surgery, including those excluded from the final review due to use of static IRT (n=26) or review format (n=20). As such, the figure reflects the broader research activity in the field rather than the final set of included studies alone.

Figure 3 depicts the total number of human and animal subjects across all IRT studies, highlighting both the growth of clinical cohorts and the continued relevance of animal models in experimental research.

**Figure 4:**
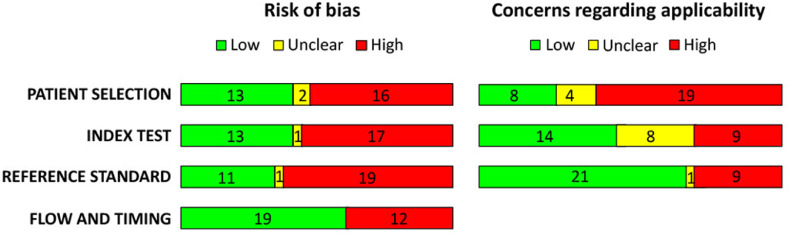
QUADAS-2 Risk of bias (left) and Applicability concerns (right). Distribution across the included studies.

Quality assessment using the QUADAS-2 tool was conducted for the 31 included studies. A summary of the methodological quality is presented in Table 2. A graphical display of the distribution of risk of bias and applicability across the included studies is presented in Figure 4.

**Table 2:** QUADAS-2 Risk of bias (left) and Applicability concerns (right). Red dots represent low risk, yellow dots respresent unclear risk, green dots represent high risk.

Study	Risk of Bias				Applicability		
	Patient Selection	Index Test	Reference Standard	Flow & Timing	Patient Selection	Index Test	Reference Standard
Chaudhry et al., 2021^16^							
Chubb et al., 2011^17^							
Chubb et al., 2013^18^							
Dagdelen et al., 2021^19^							
de la Hoz et al., 2024^20^							
de Weerd et al., 2006^21^							
de Weerd et al., 2009^22^							
de Weerd et al., 2017^23^							
Hallock et al., 2019^24^							
Ismail et al., 2024^15^							
Itoh et al., 1995^25^							
Kim et al., 2024^26^							
Ko et al., 2016^27^							
Liu et al., 2012^29^							
Meier et al., 2024^6^							
Moderhak et al., 2018^30^							
Moderhak et al., 2017^31^							
Muntean et al., 2015^32^							
Muntean et al., 2018^1^							
Nergård et al., 2017^33^							
Niepel et al., 2019^34^							
Nischwitz et al., 2021^35^							
Pereira et al., 2024^36^							
Sonda et al., 2022^37^							
Thiessen et al., 2019^38^							
Thiessen et al., 2019^39^							
Verstockt et al., 2020^40^							
Weum et al., 2016^41^							
Yassin et al., 2023^42^							
Zetterman et al., 1999^7^							
Zhu et al., 2023^43^

### Comparison with other imaging techniques

Several studies assessed the diagnostic accuracy of DIRT relative to established imaging modalities such as CTA, HHD, and indocyanine green fluorescence angiography (ICG-FA). Reported sensitivities ranged from 84 to 100 %, confirming DIRT’s reliability in identifying dominant perforators, while specificities ranged from 80 to 99 %, with false positives mainly linked to superficial veins or sensor noise.^6^^,^^18^ Intraoperative validation consistently supported DIRT’s clinical accuracy for preoperative perforator mapping.^22^, ^40^^,^^44^ Table 3 summarizes the comparative strengths, limitations, and clinical applications of DIRT and other imaging modalities.

**Table 3:** Comparative overview of imaging modalities used in perforator mapping and flap assessment.

Imaging modality	Strengths	Limitations	Optimal clinical use
CTA	High spatial resolution, detailed 3D vascular anatomy	Invasive (requires contrast and radiation), expensive	Gold standard for preoperative planning and deep vessel mapping
HHD	Portable, widely available, low cost, quick	Operator-dependent, high false-positive rate	Intraoperative confirmation of perforators
ICG-FA	High sensitivity for perfusion assessment, visualizes flow dynamics	Invasive (requires dye), transient visualization	Intraoperative perfusion validation
DIRT	Non-invasive, real-time, cost-effective	Limited spatial resolution and depth penetration	Preoperative mapping, intraoperative confirmation, and postoperative monitoring

#### CTA

CTA remains the gold standard for preoperative perforator mapping, providing detailed anatomy including vessel course, caliber, and pedicle length. Multiple studies demonstrated high concordance between CTA and DIRT, with hotspot alignment typically within 8 mm.^18^ None reported major perforators visualized on CTA being missed by DIRT.^34^^,^^41^ Sensitivities of both methods are comparable (92–94 %).^37^ Unlike CTA, DIRT is non-invasive, radiation-free, and usable intra- and postoperatively for real-time perfusion assessment.^35^^,^^36^ Its integration into surgical planning can reduce operative time by up to 1 h.^4^^,^^32^

#### HHD

HHD is widely available but highly operator dependent, with frequent false positives from deep or venous signals. DIRT correlates closely with HHD, showing PPVs up to 97 %,^6^ while providing a broader, objective surface overview. Combined use is complementary: DIRT identifies perforator sites, and HHD confirms depth and flow.^43^ This sequential approach is increasingly adopted as a practical alternative to CTA when contrast imaging is limited or contraindicated.^15^, ^24^

#### ICG-FA

ICG-FA provides high-resolution perfusion imaging but requires intravenous dye and captures only transient phases. Studies show close agreement between ICG-FA and DIRT in delineating perforasomes and perfusion zones.^16^ Although ICG-FA is more sensitive to subtle flow deficits, DIRT allows continuous, dye-free, and repeatable assessment, making it better suited for prolonged intra- and postoperative monitoring.

As summarized in Table 3, DIRT complements rather than replaces existing imaging modalities. Combined with CTA, it adds functional perfusion data to anatomical mapping; paired with HHD, it integrates surface visualization and depth confirmation. These synergistic uses underscore DIRT’s value as a versatile adjunct within multimodal imaging strategies.

### Recent advancements

This section summarizes key developments since 2018 that enhance DIRT’s capabilities, including smartphone DIRT, dominant perforator ranking, parametric flap simulations, automation and AI, and AR visualization.

#### Smartphone DIRT

**Figure 5:**
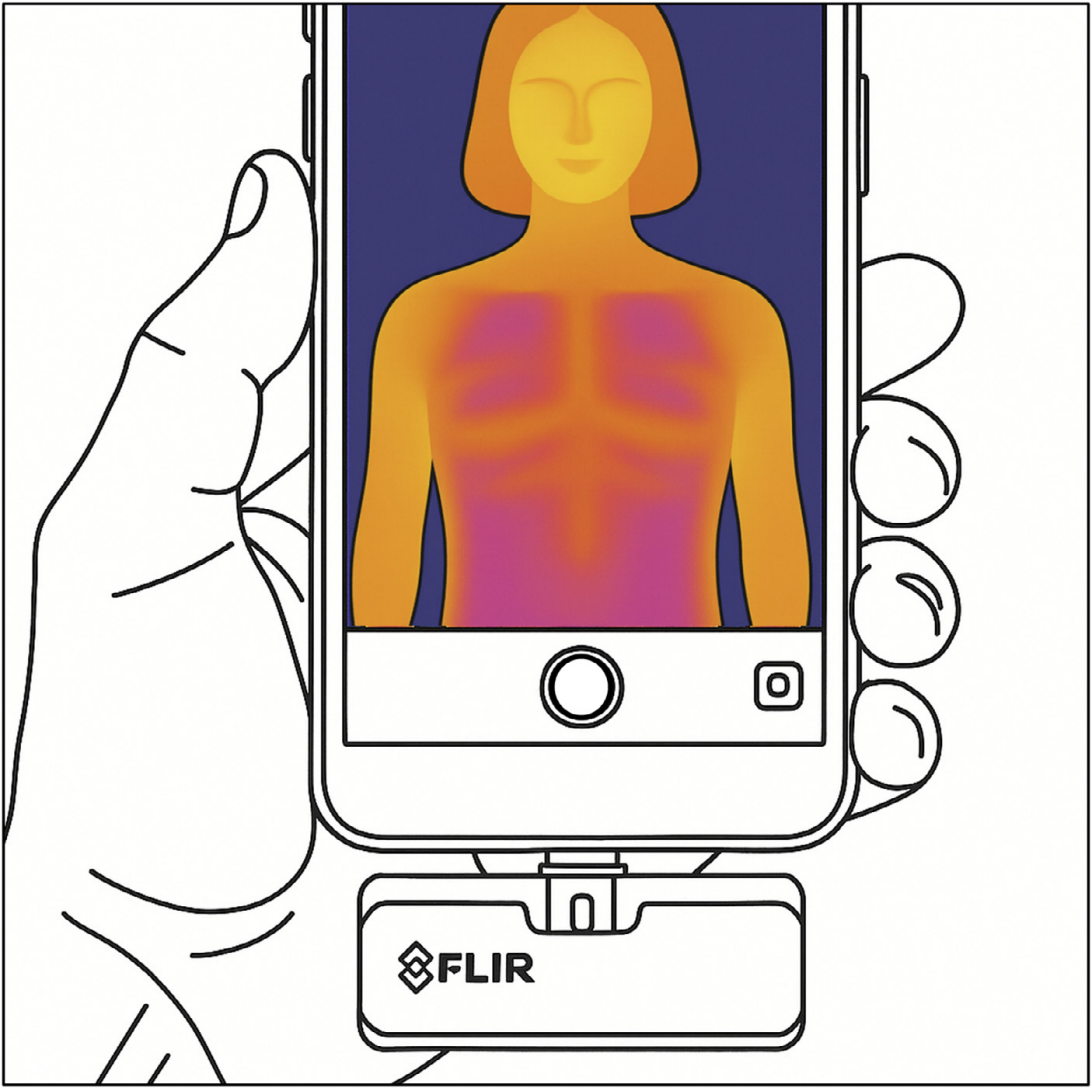
Smartphone-based DIRT using a compact FLIR sensor enables real-time visualization of cutaneous perfusion and perforator hotspots.

The advent of smartphone-compatible infrared cameras, such as the FLIR ONE Pro (Teledyne FLIR LLC, Wilsonville, OR, USA; Figure 5), has improved DIRT accessibility by enabling real-time thermal imaging via affordable mobile devices.^24^ In a cohort of 143 patients, Ismail et al.^15^ reported 83.2 % overall accuracy for smartphone-based DIRT, with sensitivity and PPV of 84.3 % and 92.9 %, respectively, compared with intraoperative visual inspection. Yassin et al.^42^ confirmed that thermal hotspots corresponded to dominant perforators and aided early detection of flap compromise. Zhu et al.^43^ found that combining smartphone DIRT with HHD improved PPV and reduced false positives, particularly in elderly patients, supporting earlier findings.^27^^,^^32^

Despite these advantages, limitations include lower spatial and thermal resolution, reduced sensitivity (approximately 100 mK vs. <50 mK for industrial cameras) in patients with thick subcutaneous tissue, and lack of advanced analysis tools such as temporal rewarming evaluation or quantitative perfusion metrics.^1^^,^^20^^,^^27^^,^^43^ Misalignment between visual and thermal images and practical issues such as battery life and ergonomics can also affect clinical reliability.^24^ Nevertheless, portability and affordability make smartphone DIRT useful for intraoperative guidance and postoperative monitoring, particularly in low-resource settings, though medical-grade systems remain preferable for preoperative planning and research.

#### Dominant perforator ranking

**Figure 6:**
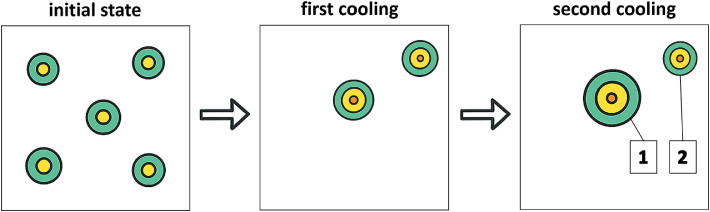
Sequential cooling–rewarming cycles allow identification of dominant perforators, characterized by earlier and more intense rewarming responses.

Muntean et al.^1^ introduced a standardized DIRT protocol for mapping perforators across donor sites using two sequential cooling–rewarming cycles. After marking hotspots in the first cycle, the second identified the hotspot that reappeared fastest and most intensely, indicating the dominant perforator with the largest perforasome. As shown in Figure 6, this approach improves perforator selection reliability. Perforators with higher flow produced earlier, more intense rewarming signals. Notably, those with oblique intramuscular paths could generate surface hotspots displaced from their fascial origin, a key anatomical nuance during flap planning.

#### Parametric flap simulations

**Figure 7:**
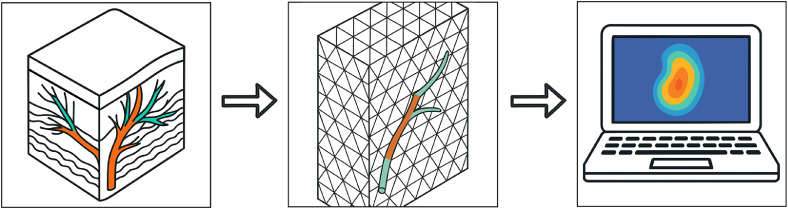
Finite element model of a DIEP flap demonstrating simulated heat transfer across layered tissue structures with embedded arterial and venous perforators.

Moderhak et al.^30^ developed a finite element DIRT simulation in COM-SOL Multiphysics (COMSOL Inc., Burlington, MA, USA). Their layered DIEP flap model included skin, adipose tissue, and embedded arterial and venous perforators (Figure 7) and compared three thermal transient parameterization methods: single-exponential, two-exponential, and simplified magnitude–temporal parametrization (SMTP). All localized perforators successfully, but SMTP provided a robust, computationally efficient alternative, producing comparable spatial contrast in under 1 s versus several minutes for the others. Simulated thermal maps closely matched those from 38 clinical DIEP cases, confirming the model’s translational relevance.^30^ By validating simulations against in vivo data, such virtual platforms support algorithm development and AI training in controlled environments.

#### Automation and artificial intelligence

To reduce observer dependence in thermal interpretation, Unger et al.^45^ developed software for automatic hotspot detection using frame-by-frame analysis of IRT videos with processing techniques such as intensity thresholding and image filtering. Their system detected dominant perforators in abdominal flaps with accuracy comparable to expert observers.^45^ This automation also enabled quantification of rewarming kinetics (e.g., time to appearance), potentially reflecting perforator quality, as discussed in Section 3.4.2.

**Figure 8:**
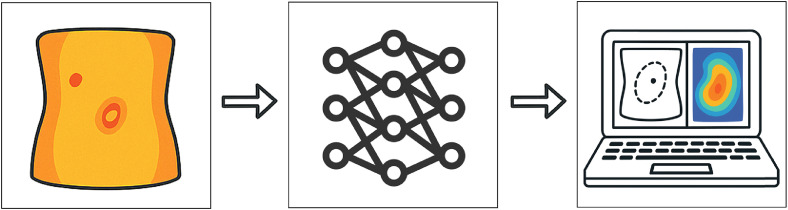
Automated IRT analysis using a CNN enables real-time segmentation and classification of thermal patterns corresponding to perforator locations.

More recently, De La Hoz et al.^20^ introduced a convolutional neural network (CNN) for real-time segmentation and perforator detection in DIRT images of DIEP flaps (Figure 8). Using annotated surgeon markings and traditional image processing for model training, the system distinguished true perforators from thermal noise and enabled automated heat-map interpretation on minimal hardware. This approach provides fast, reproducible analysis, supporting real-time decision-making in clinical practice.

#### Augmented reality visualization

A major advancement in DIRT is the integration of AR to overlay thermal data directly onto the patient. This approach addresses a limitation of conventional DIRT, where surgeons interpret thermographic images on a separate screen and manually mark perforator sites, introducing spatial error. AR enables real-time alignment of thermal data with the surgical field, improving accuracy and workflow efficiency.

**Figure 9:**
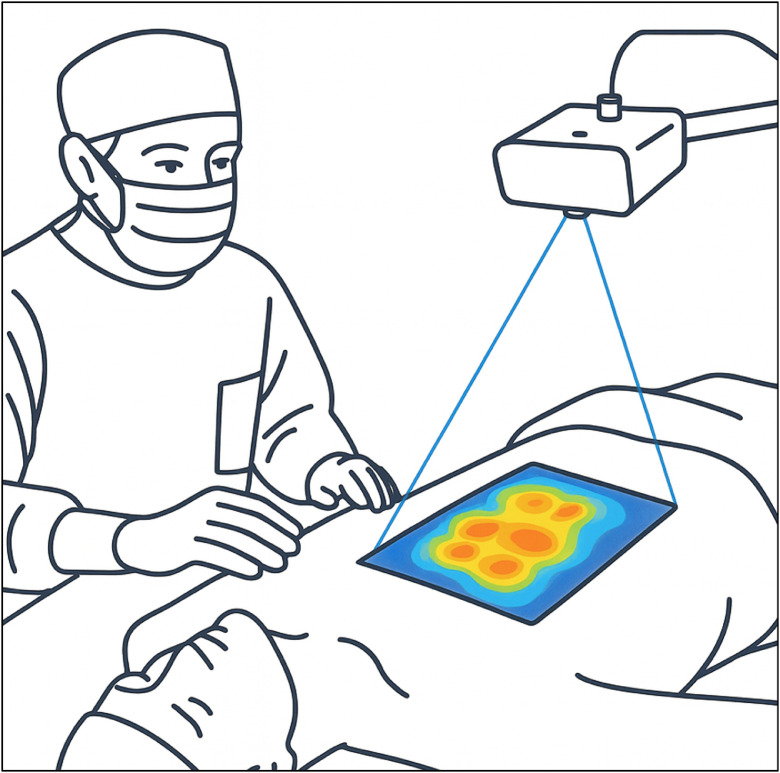
Integration of DIRT with AR allows intraoperative projection of thermal perfusion maps onto the patient’s anatomy, improving spatial accuracy during flap planning.

In 2024, Meier et al.^6^ developed a self-aligning projection system, the “Anatomy Projector.” In 50 DIEP flap patients, it projected color-coded thermal maps onto the abdomen after a standardized cooling challenge (Figure 9). Of the projected hotspots, 97.3 % corresponded to HHD signals within 1 cm, 75 % matched perforators on CTA, and among 132 dissected perforators from 71 flaps, 57 % were associated with an AR-projected hotspot. The five earliest-appearing hotspots accounted for 55 % of dominant perforators chosen during surgery, supporting Section 3.4.2. The authors concluded that AR projection of DIRT is feasible and consistent with established mapping methods, potentially enabling real-time perfusion visualization during flap elevation to support dynamic surgical decision-making.

Complementarily, Unger et al.^46^ created an AR system using the Microsoft HoloLens (Microsoft Corp., Redmond, WA, USA) to overlay thermal data as a hologram aligned with patient anatomy. The system integrated an RGB camera, depth sensor, and IR camera to generate a 3D reconstruction and superimpose thermal data accurately. In laboratory testing, mean projection error was 10 mm, adequate for orientation but suboptimal for precise perforator localization, attributed to limitations in inside-out tracking and marker technology. Despite these issues, the system achieved real-time display at five frames/s, demonstrating the feasibility of wearable AR for DIRT visualization.

Pereira et al.^36^ further combined smartphone DIRT, AR, and HHD, termed the TARDUS method, in 15 patients undergoing lower-extremity reconstruction. This approach achieved 100 % concordance in vessel location with CTA and intraoperative findings. AR overlays from preoperative CTA, displayed via smartphone, were accurately aligned and aided superficial circumflex iliac artery perforator (SCIP) flap dissection. Although antero-lateral thigh (ALT) perforator search time was not reduced by IRT alone, the integrated approach improved planning accuracy and intraoperative guidance.

## Discussion

### Clinical relevance

The findings of this review indicate that DIRT is a promising, non-invasive method for assessing skin perfusion and identifying dominant perforators. Its bedside availability and immediate feedback make it well suited to surgical workflows. DIRT can be used intraoperatively to validate or adjust preoperative plans. These advantages, together with its low cost and short acquisition time, make DIRT attractive in both high- and low-resource settings. Compared with the technically demanding and operator-dependent CDU, DIRT acquisition is relatively straightforward and yields reproducible results when standardized cooling and camera positioning are applied.

For effective clinical implementation, DIRT should be integrated into established microsurgical workflows. Standard operating procedures should include (1) standardized preoperative cooling and imaging for perforator mapping, (2) intraoperative validation after flap elevation, and (3) postoperative surveillance for vascular compromise. Integration into these phases adds minimal time, typically 5–10 minutes per phase, and can be performed by trained surgical staff using portable infrared systems.

Training is essential but straightforward: reports describe a short learning curve of fewer than five supervised cases for consistent interpretation.^24^^,^^39^ Training should emphasize recognition of rewarming patterns, differentiation of artifacts, and correlation of hotspots with anatomical landmarks, ideally within standardized protocols aligned with published SOPs^38^ to ensure reproducibility and enable multicenter comparison.

### Comparison to existing techniques

The main limitation of DIRT is its inability to depict subsurface anatomy, including perforator trajectories and branching. Therefore, CTA remains the gold standard in many centers for DIEP planning, as it provides detailed three-dimensional visualization of pedicle anatomy. In contrast, DIRT shows only terminal perfusion at the skin surface. Yet the surgical objective is not merely anatomical mapping but locating the skin site where perforator dissection ensures adequate flap perfusion. DIRT addresses this directly by highlighting the fastest rewarming zones, offering immediate functional insight, while CTA provides an indirect anatomical estimate based on vessel size and course. Several authors have proposed a complementary approach combining CTA’s structural detail with DIRT’s physiological assessment. Although CTA excels in deep vascular mapping, DIRT shows comparable diagnostic accuracy for dominant perforators, and some centers have substituted CTA with combined DIRT and HHD without loss of outcome. Modality choice ultimately depends on institutional resources and surgeon preference. Compared with HHD, DIRT offers a broader, more objective field of view and is less likely to miss relevant perforators because of limited scanning areas. Compared with ICG-FA, DIRT shows similar perfusion concordance^16^ while avoiding invasiveness and intravenous contrast.

### Latest advancements

DIRT technology is evolving rapidly. Portable infrared cameras compatible with smartphones have expanded clinical accessibility and show strong diagnostic performance, especially when combined with HHD.^43^ Although their resolution is lower than medical-grade systems, affordability and portability make them attractive for outpatient and intraoperative use. Integration with augmented reality (AR) platforms improves interpretation by projecting hotspot maps directly onto the patient’s skin, enhancing spatial accuracy and reducing cognitive workload.^6^ Current projection accuracy remains around 10 mm, but ongoing developments indicate strong potential for intraoperative navigation.

DIRT has progressed from simple hotspot detection to quantitative assessment, using rewarming kinetics to rank dominant perforators by functional capacity. This shift parallels surgical advances emphasizing physiological over purely anatomical planning. Recent work demonstrates automated hotspot segmentation with convolutional neural networks (CNNs), reducing interobserver variability and enabling standardized, reproducible analysis.^20^ Together, these innovations suggest DIRT is nearing routine clinical integration, though widespread adoption still requires further standardization and validation.

### Risk of bias

Methodological quality and applicability of the included studies were assessed using the QUADAS-2 tool (Table 2, Figure 4). Main concerns involved patient selection, index test, and reference standard domains. Flow and timing issues mainly occurred in studies comparing preoperative DIRT with earlier CTA.

For patient selection, most studies enrolled consecutive or convenience samples without stratification for demographic or clinical variables. This broad inclusion improves clinical relevance but limits analysis of potential confounders. Zhu et al. restricted inclusion to patients without cardiovascular risk factors,^43^ which reduced applicability.^47^ Many studies were single-center with modest sample sizes (9–143 participants; only one >100^15^), increasing selection bias risk.

The highest bias risk occurred in the index test domain. In most reports, DIRT was interpreted after the standard-of-care test (CTA, Doppler, or intraoperative inspection), so blinding was unclear or absent, potentially overestimating diagnostic accuracy. Few studies prespecified objective thresholds or detailed cooling protocols, resulting in uncertain reproducibility.

The reference standard domain was also problematic. HHD, a common comparator, is operator dependent and not firmly validated. Concordance between DIRT and HHD may therefore overstate DIRT performance. In studies using CTA, imaging was often performed at different times and under different physiological conditions (outpatient vs. intraoperative), introducing additional flow and timing bias due to temperature and medication effects.

Overall, the QUADAS-2 assessment indicates that variability in reported diagnostic performance of DIRT largely reflects study-level bias in these domains.

### Limitations

This review has several limitations. Considerable heterogeneity existed among included studies in design, reference standards, and DIRT protocols, which limited reproducibility and direct comparison of diagnostic accuracy. Different comparators were used across perioperative phases (CTA preoperatively, HHD or direct visualization intraoperatively, and clinical evaluation postoperatively). Technical variability in cooling methods, ambient conditions, and camera specifications further reduced consistency. Although standardization efforts have been published,^5^^,^^39^ no universally accepted DIRT protocol exists.

Patient selection and clinical context also varied widely, ranging from animal models and healthy volunteers to patients undergoing DIEP, ALT, SCIP, or TRAM flap procedures. Differences in cooling temperature, exposure duration, rewarming time, and analytical approach (from visual hotspot detection to AI-based segmentation) limited cross-study comparability and interpretation. Reference standards differed as well (CTA, HHD, ICG-FA, intraoperative observation), each with distinct strengths and biases, further affecting generalizability and underscoring the need for standardized validation frameworks.

Methodological variability and inconsistent outcome reporting precluded quantitative meta-analysis. Although some studies reported sensitivity, specificity, PPV, and NPV, heterogeneity in design, protocols, and comparators prevented data pooling. Many lacked extractable contingency data, so this review provides a qualitative synthesis reflecting current methodological diversity.

Where quantitative data were available, DIRT showed high diagnostic performance (sensitivity 84–100 %, specificity 80–99 %, PPV 67–97 %, NPV 74–98 %), but these estimates stem from methodologically diverse studies using non-uniform effect measures^6^^,^^15^^,^^18^^,^^32^^,^^35^^,^^37^^,^^44^^,^^43^ and should be interpreted cautiously.

Publication bias cannot be excluded, as negative or inconclusive results may be underrepresented in this rapidly evolving field. Finally, narrative synthesis may introduce interpretation bias, particularly as many studies remain exploratory and focus on technical feasibility rather than standardized clinical outcomes.

### Future research

To support broader clinical adoption, several research priorities must be addressed. Standardization is paramount, as current literature shows wide methodological variation that hinders comparison. Development of consensus guidelines, similar to those for CTA, is essential. Comparative studies should define optimal parameters to improve reproducibility and diagnostic accuracy.

Prospective trials are needed to confirm clinical efficacy. Although early evidence suggests DIRT may match CTA in perforator identification, noninferiority studies assessing flap survival, complications, and operative duration are lacking. Trials evaluating DIRT for postoperative monitoring could clarify its role in early detection of flap compromise, while cost-effectiveness studies would further support implementation.

Integration of DIRT with digital technologies, particularly AI and AR, represents a key future direction. CNN-based models^20^ and simulation tools^30^ may enable standardized, operator-independent workflows. Training AI on simulated thermal responses could streamline model development and enhance scalability.

AR systems, such as those by Meier et al.,^6^ offer promising visualization by projecting hotspot maps intraoperatively, though current limitations in accuracy and latency remain. Future studies should evaluate AR-guided planning regarding learning curves, surgical efficiency, and decision-making impact. The convergence of AR and AI may permit real-time overlay of both anatomical and algorithmically prioritized perforators, improving intraoperative navigation.

These advances mark a shift from standalone imaging to integrated, intelligent surgical ecosystems. Continued innovation should emphasize clinical integration to optimize workflow, precision, and patient outcomes.

## Conclusion

This systematic review provides an overview of the current state and recent advancements in the use of DIRT within free flap surgery. The collected literature demonstrates that DIRT offers high sensitivity and clinical utility for perforator selection and perfusion assessment during preoperative planning, intraoperative guidance, and postoperative monitoring. Its non-invasive nature, real-time feedback, and capacity for repeated evaluations without adverse effects to the patient position DIRT as a promising complementary modality to established techniques such as CTA and HHD, and a potential alternative in selected cases.

Although DIRT shows strong potential as a complementary, non-invasive modality in reconstructive microsurgery, its widespread clinical implementation remains contingent upon further standardization and validation. Recent technological developments, including integration with AR, AI-based automation, and smartphone-compatible systems, have markedly improved its practicality and diagnostic performance. However, multicenter, largescale studies are still required to confirm its reproducibility and clinical impact across diverse settings. Therefore, DIRT should presently be regarded as a promising adjunct technique that is approaching clinical maturity, rather than one that is fully ready for broad adoption.

## Author contributions

Conceptualization, Clarys W., Hotome V., Thiessen F.; methodology, Clarys W., Hotome V., Thiessen F., Hummelink S.; investigation, Clarys W., Hotome V.; data curation, Clarys W., Hotome V., Thiessen F.; writing (original draft preparation), Clarys W.; writing (review and editing), Clarys W., Verspeek S., Hummelink S., Thiessen F., Steenackers G.; visualization, Clarys W., Hotome V.; supervision, Verspeek S., Verhoeven V., Tjalma W.A.A., Steenackers G., Thiessen F. All authors have read and agreed to the published version of the manuscript.

## Funding

This research has been funded by Research Foundation-Flanders (FWO) under a Strategic Basic Research (SB) fellowship 1SB9825N (Warre Clarys), FWO TBM Research Project, “The use of dynamic infrared thermography for perforator mapping and quality improvement in autologous breast reconstructions” (project number 41882 (FWO TBM FN7023).

## Data availability

Extracted data supporting the findings of this review are available from the corresponding author upon reasonable request. No analytic code was used, as no statistical meta-analysis was performed.

## Consent for publication

The principles outlined in the Declaration of Helsinki have been followed and written informed consent was obtained from the patient for publication of this communication and the accompanying images.

## Ethical approval

the authors are accountable for all aspects of the work in ensuring that questions related to the accuracy or integrity of any part of the work are appropriately investigated and resolved. Ethical approval has been obtained from ethics committee. Belgian registration: B3002024000116.

## Declarations of competing interest

The authors declare that they have no conflicts of interest.
